# Allelic variants in the PHTF1-PTPN22, C12orf30 and CD226 regions as candidate susceptibility factors for the type 1 diabetes in the Estonian population

**DOI:** 10.1186/1471-2350-11-11

**Published:** 2010-01-20

**Authors:** Konstantinos Douroudis, Kalle Kisand, Virge Nemvalts, Tarvo Rajasalu, Raivo Uibo

**Affiliations:** 1 Immunology group, Institute of General and Molecular Pathology, University of Tartu, Tartu, Estonia; 2 Department of Internal Medicine, Kuressaare Hospital, Kuressaare, Estonia; 3 Department of Internal Medicine, University of Tartu, Tartu, Estonia

## Abstract

**Background:**

Type 1 diabetes is a multifactorial disease with a strong genetic component. The aim of the study was to assess the impact of single nucleotide polymorphisms (SNPs) in several genes as susceptible markers in the risk of type 1 diabetes in the Estonian population. Methods: The rs6679677 (1p13), rs17696736 (12q24) and rs763361 (18q22) were genotyped in a total of 230 controls and 154 type 1 diabetes patients of Estonian origin.

**Results:**

The rs6679677 A (OR = 2.13, 95%CI = 1.48-3.08, p = 0.00001), rs17696736 G (OR = 1.53, 95%CI = 1.14-2.04, p = 0.0046) and rs763361 T (OR = 1.48, 95%CI = 1.11-1.98, p = 0.0084) alleles were associated with risk of type 1 diabetes.

**Conclusions:**

The current study supports the rs6679677 (PHTF1-PTPN22), rs17696736 (C12orf30) and rs763361 (CD226) SNPs as susceptibility factors for type 1 diabetes outside the major histocompatibility region (MHC) region. The full study had 80% or above to detect an odds ratio of 1.8 under the assumption of an additive model at type 1 error rate, α = 0.05.

## Background

The genetic predisposition to type 1 diabetes is strongly linked to MHC region [1] but non - MHC locus associated genes are also considered to predispose in the risk of type 1 diabetes. Evidence strongly supports the insulin gene [2], the CTLA-4 locus [3] and the PTPN22 gene [4] as important candidates outside the MHC region. Meanwhile, the established genetic associations with type 1 diabetes explain roughly half of the genetic risk for type 1 diabetes, indicating that other loci exist. In favor of it, genome wide association studies have recently reported new chromosomal regions and novel candidates in the risk of type 1 diabetes [5,6]. Of note, within the CD226 gene (18q22) the rs763361 SNP was found to be associated with type 1 diabetes [5]. The CD226 gene is expressed on the majority of immune cells including natural killer (NK) cells and T cells mediating their activation and differentiation [7]. Moreover, the rs17696736 and the rs6679677 in the chromosomal regions of 12q24 and 1q13 respectively, were also reported to be associated with type 1 diabetes [8]. In particular, the rs17696736 SNP (C12orf30 gene) is located within a large linkage disequilibrium (LD) block that contains several genes of possible function relevance to type 1 diabetes [8]. The rs6679677 SNP is an intergenic SNP located downstream of the round spermatid basic protein 1 (RSBN1) and in the promoter site of the putative homeodomain transcription factor 1 (PHTF1) gene. The complexity of type 1 diabetes, the heterogeneity of allele frequencies across populations and the difficulties to localize the true causative variants within a LD block recommends the replication analysis of reported SNPs in order to establish their impact on individual ethnic groups. In the current study we sought to replicate recently reported associations from the genome wide association (GWA) studies in order to evaluate their impact as possible candidates in the risk of type 1 diabetes in Estonian population.

## Methods

### Subjects

An ethnically homogenous population of Estonian origin including 230 controls (mean age 45.9 ± 14.5 years, 139 females) and 154 type 1 diabetes patients (mean age at diagnosis 22.0 ± 14.3 years, 77 females) were enrolled into a case-control study. Estonia is situated in the north of Europe, on the eastern shores of the Baltic Sea and Estonians belong to the Finno-Ugric ethnic group. Of note, our population cohort is originated from Saaremaa County in Estonia. Saaremaa is the biggest island of Estonia where the population has been relatively permanent over generations compared to the major mainland regions in Estonia. All patients with type 1 diabetes were diagnosed according to the criteria established by the Expert Committee on the Diagnosis and Classification of Diabetes Mellitus [9]. The Ethics Committee of the University of Tartu approved the study, and informed consent was obtained from all participants.

### Genotyping

The genotyping of rs6679677 (PHTF1-PTPN22), rs17696736 (C12orf30) and rs763361 (CD226) SNPs was carried out using the SNP genotyping assay (Applied Biosystems, Foster City, CA). PCR reactions were run on the ABI 7000 instrument (Applied Biosystems) using the following cycling parameters: after the first step at 95°C for 10 min, 40 cycles of denaturation at 92°C for 15 s and extension at 60°C for 1 min and the genotypes were analysed using the allelic discrimination function of the instrument. A 100% genotyping success rate was obtained for all studied SNPs.

### Statistical analysis

The R (version 2.4.1) statistical computer program (The R Foundation for Statistical Computing, Boston, MA, http://www.r-project.org) was employed to obtain odds ratio (OR) values as well as Wald's confidence intervals (CI) for alleles and genotypes. Hardy-Weinberg equilibrium (HWE) was performed using the SHEsis software [10]. Permutation analysis was performed using the Haploview 3.2 version software [11]. 10000 permutations were carried out to estimate the significance of the results, correcting for the three loci tested. Power calculations were performed using the PS software [12]. A p value of < 0.05 was considered significant for all analyses.

## Results

The rs6679677, rs17696736 and rs763361 SNPs were genotyped in all studied subjects and the analysis of genotypes distribution showed no significant deviation from the HWE in type 1 diabetes or in the control group (p > 0.05). A significant higher frequency of the rs6679677 AA (OR = 4.23, 95%CI = 1.56-11.52, p = 0.0047), rs17696736 GG (OR = 2.32, 95%CI = 1.25-4.28, p = 0.0074) and rs763361 TT (OR = 2.29, 95%CI = 1.25-4.18, p = 0.0071) genotype was observed in the group of type 1 diabetes patients when compared to controls (table 1). Moreover, the distribution frequency of the rs6679677 A (OR = 2.13, 95%CI = 1.48-3.08, p = 0.00001), rs17696736 G (OR = 1.53, 95%CI = 1.14-2.04, p = 0.0046) and rs763361 T (OR = 1.48, 95%CI = 1.11-1.98, p = 0.0084) allele was significantly higher in type 1 diabetes when compared with the control group (table 1). Power calculation analysis showed that given the frequencies and genotype odds ratio observed in our population for the homozygotes (high risk alleles), our case control study had 85%, 89% and 90% power (at α = 0.05), to detect significant differences for the rs6679677, rs1769676 and rs763361 markers, respectively. Of note, all observed type 1 diabetes associations with the rs6679677 A, rs17696736 G and rs763361 T alleles remained significant after permutation analysis (10000 permutations, giving p = 0.0004, p = 0.016 and p = 0.034, respectively).

**Table 1:** Genotype and allele frequencies of the rs6679677, rs17696736 and rs763361 SNPs in the type 1 diabetes patients and control subjects.

SNP (Gene region)	Cases (%) (N = 154)	Control(%) (N = 230)	Odds ratio (95%CI)	P
**rs6679677 ** **(PHTF1-PTPN22)**				
AA	13(8.4)	6(2.6)	4.23(1.56-11.52)	0.0047
AC	53(34.5)	52(22.6)	1.99(1.26-3.16)	0.0034
CC	88(57.1)	172(74.8)	1*	
A	79(25.6)	64(13.9)	2.13(1.48-3.08)	0.00001
C	229(74.4)	396(86.1)	1*	
**rs17696736** **(C12orf30)**				
AA	38(24.7)	88(38.3)	1*	
AG	83(53.9)	109(47.4)	1.76(1.10-2.84)	0.0194
GG	33(21.4)	33(14.3)	2.32(1.25-4.28)	0.0074
A	159(51.6)	285(61.9)	1*	
G	149(48.4)	175(38.1)	1.53(1.14-2.04)	0.0046
**rs763361** **(CD226)**				
CC	36(23.4)	76(33.1)	1*	
CT	79(51.3)	118(51.3)	1.41(0.87-2.30)	0.16
TT	39(25.3)	36(15.6)	2.29(1.25-4.18)	0.0071
C	151(49.1)	270(58.7)	1*	
T	157(50.9)	190(41.3)	1.48(1.11-1.98)	0.0084

1*: referent estimate

## Discussion

In the present study we attempted to assess the impact of novel reported findings from genome wide type 1 diabetes association studies in a population of Estonian origin. In favor of it, the association of the rs6679677 SNP in susceptibility of type 1 diabetes has been confirmed; thus making it an additional candidate within the 1q13 region. Notably, the rs6679677 SNP is located in the same haplotype block with the rs2476601 SNP of the PTPN22 gene. Having previously demonstrated a significant association of T allele of rs2476601 SNP in type 1 diabetes [13], we sought to assess the hypothesis that the rs2476601 SNP might not be the sole causal variant in the region. However, according to our previous findings [13] and based on the genetic data alone, we were unable to distinguish between the two studied SNPs since they are in perfect LD (R^2 ^= 1.00, D' = 1.00). This is in correlation with the results of recent studies in which an association of the rs6679677 SNP with type 1 diabetes has also been reported and the effects of the two SNPs could not be distinguished due to tight LD [14,15]. Moreover, through using the Genomatix web tool for SNP analysis http://www.genomatix.de, as Qu et al. demonstrated [15], we revealed that the substitution of cytosine (C) to adenine (A) in the rs6679677 locus predicts the loss of two (CAAT, CDP) and the generation of one (Sox-5) transcription binding sites. In addition we agree with Qu et al. [15] that the region around PTPN22 may harbor additional disease related alleles not yet found.

Furthermore, we confirmed that the rs763361 SNP in the CD226 gene was associated with susceptibility to type 1 diabetes. The rs763361 SNP is predicted to be a functional SNP, residing in the final 3' of the exon 7 and alters the exon splicing silencer (ESS) sequence [5]. The disease-susceptible minor allele A, which encodes Ser307, maintains the wild type ESS sequence and compared to the resistant Gly307 allele would have a higher silencer function that reduces the productive splicing of exons 6 and 7. Notably, the CD226 molecule is expressed on a variety of hematopoietic cells and interaction with its ligands poliovirus receptor (PVR, CD155) and Nectin-2 (CD112) results in a variety of cellular responses of innate and adaptive immunity [16]. Recently, new studies have provided additional evidence and support the role of rs763361 SNP in the risk of autoimmune thyroid disease [17], rheumatoid arthritis, multiple sclerosis [18,19] and Wegener's granulomatosis [19].

Moreover, we showed that the rs17696736 SNP in the C12orf30 gene also contribute to susceptibility of type 1 diabetes. Interestingly, the C120rf30 gene maps into a region of extensive linkage disequilibrium including several genes that represent functional candidates of type 1 diabetes-susceptibility because of their presumed roles in immune signaling. Remarkably, the SH2B3 (SH2B adaptor protein 3), TRAFD1 (TRAF-type zinc finger domain containing 1) and PTPN11 (protein tyrosine phosphatase, non-receptor type 11) map on the same region with the C12orf30 gene. Therefore, for this region in particular, extensive resequencing, further genotyping and targeted functional studies will be essential steps in identifying which gene, or genes, are causal.

One common issue in the study of complex diseases, including type 1 diabetes, is the limited sample size, resulting in adequate power to detect association or the risk of false positive results. Typically, the full study had 80% power or above to detect an odds ratio of 1.8 under the assumption of an additive model at type 1 error rate, α = 0.05. Further, take in consideration the sample size of our study, permutation analysis has been applied in order to evaluate the impact of our findings. Of note, all the reported associations remained significant after applied permutation analysis.

Noticeably, further genotyping, resequencing and fine mapping could assist in clarifying the true causative genes within a strong LD block and could reveal new susceptible allelic variants for type 1 diabetes. The potential of the latter approaches to detect new associations have recently been demonstrated by Nejentsev et al [20]. Moreover, we should also take into account that due to allelic heterogeneity, there is a variation in risk allele frequencies between populations, which may account for differences in disease prevalence between ethnic groups.

## Conclusion

In the current study, we have demonstrated a significant association between recently reported SNPs and type 1 diabetes in Estonian population. To confirm our findings, further associated studies are needed to elucidate the functional role of these SNPs as likely mediators for autoimmunity and as potential therapeutic targets for antidiabetic drugs. The probability that the newly reported loci with type 1 diabetes may predispose other autoimmune diseases should also be examined.

## Competing interests

The authors declare that they have no competing interests.

## Authors' contributions

KD performed the genotyping, the statistical analysis, interpreted the data and drafted the manuscript. KK, TR and VN participated in materials' and data collection. RU participated in the initiation and coordination of the study. All authors read and approved the final manuscript.

## Pre-publication history

The pre-publication history for this paper can be accessed here:

http://www.biomedcentral.com/1471-2350/11/11/prepub
